# The Impact of Pedagogical Agents' Gender on Academic Learning: A Systematic Review

**DOI:** 10.3389/frai.2022.862997

**Published:** 2022-06-20

**Authors:** Marjorie Armando, Magalie Ochs, Isabelle Régner

**Affiliations:** ^1^Aix Marseille Univ, CNRS, LIS UMR 7020, Marseille, France; ^2^Aix Marseille Univ, CNRS, LPC, Marseille, France; ^3^Pôle pilote Ampiric, Institut National Supérieur du Professorat et de l'Éducation, Aix-Marseille Université, Marseille, France

**Keywords:** virtual agent, gender, pedagogical agent, learning environment, gender stereotypes, systematic review

## Abstract

Virtual learning environments often use virtual characters to facilitate and improve the learning process. These characters, known as pedagogical agents, can take on different roles, such as tutors or companions. Research has highlighted the importance of various characteristics of virtual agents, including their voice or non-verbal behaviors. Little attention has been paid to the gender-specific design of pedagogical agents, although gender has an important influence on the educational process. In this article, we perform an extensive review of the literature regarding the impact of the gender of pedagogical agents on academic outcomes. Based on a detailed review of 59 articles, we analyze the influence of pedagogical agents' gender on students' academic self-evaluations and achievements to answer the following questions: (1) Do students perceive virtual agents differently depending on their own gender and the gender of the agent? (2) Does the gender of pedagogical agents influence students' academic performance and self-evaluations? (3) Are there tasks or academic situations to which a male virtual agent is better suited than a female virtual agent, and vice versa, according to empirical evidence? (4) How do a virtual agent's pedagogical roles impact these results? (5) How do a virtual agent's appearance and interactive capacities impact these results? (6) Are androgynous virtual agents a potential solution to combatting gender stereotypes? This review provides important insight to researchers on how to approach gender when designing pedagogical agents in virtual learning environments.

## 1. Introduction

Pedagogical agents are virtual characters in digital environments used to improve learning in educational settings (Mohtadi et al., 2014; Schroeder et al., 2017). They can take on different roles, such as expert, mentor, or motivator (Baylor and Kim, 2005). As shown in a meta-analytic review of 43 studies by Schroeder et al. (2013), pedagogical agents can have a positive effect on students' free recall ability, knowledge retention, and transfer of prior knowledge to new situations or problems. However, some characteristics of pedagogical agents may impact the learning process: for instance, how realistic the virtual agents' appearance is Baylor and Kim (2004), the way they communicate with learners, verbally or nonverbally, positively or negatively (Gratch et al., 2007; Pecune et al., 2016), or the way they deliver feedback, using voice, text, or both (Kim and Baylor, 2016).

Virtual agents' gender is another feature that users can perceive from the agents' appearance (Lee, 2003). Yet few studies have evaluated the impact of pedagogical agents' gender, which is surprising considering the amount of research in Social Cognition documenting the impact of the gender of both learners and teachers on academic learning. Social Cognition and human-to-human studies are particularly interesting in the domain of virtual agents, as research shows that individuals have a propensity to interact with virtual agents as if they were human (Nass and Moon, 2000). Research in Social Cognition and Cognitive Psychology can, therefore, be enlightening for understanding users' perception of virtual characters and the effect of these perceptions on their performance. This is why we present some major Social Cognition research on the impact of learners and teachers' gender on learners' academic outcomes. For instance, Sansone (2019) conducted a survey on the link between high school students' beliefs about women's abilities in math and science and their teacher's gender, finding that students were less likely to report that men are better than women in math/science when assigned to female teachers. Teachers' behavior can also impact girls' and boys' learning differently: a large scale survey conducted by Forgasz and Leder (1996) showed that students who perceived their math teachers to be interested in them as individuals were more likely to have functional beliefs about themselves in mathematics, and this was more critical for female learners than male learners. Core beliefs represent general and strongly held views about ourselves, others, and the world; they influence the way we react in different circumstances. Functional beliefs are rational thought patterns that are generally useful for individuals to achieve their goals (Ellis, 1962). In the forementioned study, math teachers' behaviors seemed to favor boys over girls: boys had more interactions with their teachers, teachers were more tolerant of boys' misbehavior, and they had higher expectations of boys (Forgasz and Leder, 1996). A meta-analysis conducted by Lindberg et al. (2010) from 242 studies published between 1990 and 2007 indicated that while male and female learners performed similarly in mathematics, female students reported higher anxiety, more discomfort, and lower interest and self-efficacy in math classes than male students. Parents themselves tend to attribute different explanations for their children's academic performance depending on their gender: they explain their sons' mathematical success as due to their natural talent, whereas they explain their daughters' as due to their effort (Yee and Eccles, 1988). These results were replicated by Räty et al. (2002) who also found that parents of boys evaluated their child's mathematical competence as higher than parents of girls, and parents of girls perceived them as surpassing boys in reading. Despite this, parents still attributed competence in reading as resulting from the effort of girls but to the natural talent of boys. By explaining their daughters' success in math as due to effort, the authors suggested that parents may undermine both their own and their daughters' estimation of their daughters' success in mathematics, hence raising possible doubts about their future success in a domain that they think gets increasingly complicated; meanwhile, they may encourage boys to develop greater confidence in their future success (Yee and Eccles, 1988).

All these differences reflect the influence of gender stereotypes that lead people to consider men to be better at math than women, and women to be better in liberal arts -such as literature, e.g.,- than men. In addition, studies have shown that the fear of being negatively stereotyped in a skill area produces negative thoughts, which in turn reduce individuals' working memory capacity and impair learning and performance (Schmader and Johns, 2003). This phenomenon, called *Stereotype Threat* (Steele and Aronson, 1995), applies to different stereotypes and social groups, such as boys in reading tests (Pansu et al., 2016) and girls and women in math tests (Régner et al., 2014). The effects of Stereotype Threat can be reduced using different strategies, such as reading a story about a successful role model before taking a test (Bagès and Martinot, 2011; Bagès et al., 2016).

Presenting pedagogical agents as role models could be a potential solution for reducing the effects of Stereotype Threat. Researchers designing agents should take into account the gender of both learners and pedagogical agents to adapt the agent to the learners. The advantages of adapting virtual agents to participants have been demonstrated in several studies. For instance, in Vilaro et al. (2021), participants (all Black women) liked Black female agents for being artificial, hence creating a sense of trust and freedom where participants could avoid inherent biases and racism. In virtual learning environments, research has shown the impact of virtual agents' gender on human-agent interactions (refer to Section 3.4). However, the gender of *pedagogical* virtual agents is rarely considered an important characteristic in the design of virtual learning environments, whereas most pedagogical agents are human-like, and their gender can have an impact on academic outcomes (refer to Section 3.5). In terms of perception, various studies have shown that male virtual agents are rated as more powerful (Nunamaker et al., 2011), more expert (Nunamaker et al., 2011), and more knowledgeable (Baylor and Kim, 2004), whereas female agents are rated as more likable (Nunamaker et al., 2011) and more attractive (Lunardo et al., 2016). These attributes are important in learning environments, as competent and expert agents improve learners' performance (Baylor and Kim, 2004), and likable and attractive agents improve learners' self-perception including their self-efficacy (feeling of achievement) (Rosenberg-Kima et al., 2008), which may help improve their performance (Plant et al., 2009).

In this article, we present an extensive state of the art focusing on the *effects of pedagogical agents' gender in virtual learning environments*. We explore the impact of gender on the users' perceptions of agents and on their learning.

This article is organized as follows. In the next section, we explain our methodology used to conduct the state of the art and particularly how we used the PRISMA method to select relevant articles (Webster and Watson, 2002). In Section 3, the selected articles are summarized in Tables 1, 2 to provide a comprehensive review of research on the impact of pedagogical agents' gender on learners' performance and self-perception in academic domains. We discuss the articles summarized in the tables in Section 3.4 and Section 3.5. In Section 3.4, we address research highlighting the impact of virtual agents' gender on users' perceptions. In Section 3.5, we focus on pedagogical agents and the impact of their gender on learners' academic outcomes. The last section discusses what could be done in future research on virtual learning environments to reduce gender stereotypes and improve learners' performance, and the important research questions that arise from this review.

**Table 1:** Summary of articles on perceptive studies of virtual agents depending on their gender, regardless of the application domain.

**Reference(s)**	**Agent(s)**	**Participant(s)**	**Task(s)**	**Measure(s)**	**Result(s)**
Lee (2003)	• 1 MA • 1 FA • 2-D • Text • Cartoon • Adviser	• 28 MP • 88 FP • avg age N/A	Playing a multiple-choice game with an agent. Participants could change their answer after they were told the agent's answer. It was specified that the agent's answer might not be correct.	• Masculinity • Attractiveness • Competence • Trustworthiness • Persuasiveness (sport or fashion questions)	• *Masculinity*: MA > FA • *Attractiveness*, *competence*: FA > MA • *Persuasiveness (sport)*: MA > FA • *Persuasiveness (fashion)*: FA > MA
Zanbaka et al. (2006)	• 2 MA • 2 FA • 3-D • Voice • Realist • Speaker	• 41 MP • 97 FP • avg age 20.6	Listening to agents deliver a message to change participants' attitudes about university-wide comprehensive exams.	• Persuasiveness	• *Persuasiveness*: - MP: FA > MA - FP: MA > FA
Guadagno et al. (2007)	• 1 MA • 1 FA • 1 neutral • 3-D • Voice • Realist • Speaker	• 37 MP • 29 FP • avg age N/A	Listening to agents talk about changes to university security policy.	• Likeability • Credibility • Presentation quality • Persuasiveness	• *Likeability, credibility*, *presentation quality*: - MP: not significant - FP: FA > MA • *Persuasiveness*: - MP: MA > FA - FP: FA > MA
Guadagno et al. (2007)	• 1 MA • 1 FA • 3-D • Voice • Realist • Speaker	• 85 MP • 89 FP • avg age N/A	Listening to agents talk about changes to university security policy.	• Likeability • Credibility • Presentation quality • Social presence • Persuasiveness	• *Likeability*: FA > MA • *Credibility, presentation quality*: not significant • *Persuasiveness*: - MP: MA > FA - FP: not significant
Gulz et al. (2007)	• 2 MA • 2 FA • 2-D • Voice • Realist • Presenter	• 72 MP • 86 FP • avg age N/A	Listening to agents present university program engineering.	• Favorite agent • Interest	• *Favorite agent*: - MP: less feminine and less masculine agents > more masculine agent > more feminine agent - FP: less feminine and less masculine agents > more feminine agent > more masculine agent • *Interest*: - MP: more feminine and more masculine agents > less feminine and less masculine agents - FP: more femine and more masculine and less feminine agents > less masculine agent
Dill et al. (2008)	• 16 MA • 16 FA • 3-D • Realist • Video game characters	• 61 MP • 120 FP • avg age 18.82	Watching a PowerPoint presentation opposing still pictures of video game characters and male or female US senators. Reading a real-life story about the sexual harassment of a female student by a male professor.	• Tolerance for sexual harassment • Rape-supportive attitudes	• *Tolerance for sexual* *harassment*: MP > FP • *Rape-supportive attitudes*: MP > FP
Rosenberg-Kima et al. (2008)	• 4 MA • 4 FA • 3-D • Voice • Realist • Speaker	• 111 FP • avg age 19.72	Listening to an agent describe four female engineers and the benefits of engineering, with or without the agent present.	• Interest • Self-efficacy • Utility for engineering • Fewer engineering gender stereotypes	• Self-efficacy and Interest in engineering: young and cool agents > other agents • *Utility for engineering*: MA > FA (not significant) • *Fewer engineering* *gender stereotypes*: FA > MA
Niculescu et al. (2009)	• 3 MA • 3 FA • 1 neutral • 3-D • Voice • Cartoon • Assistant	• 24 MP • 24 FP • avg age N/A	Interacting with agents about medical queries, evaluating an androgynous agent's gender either after or before rating non-androgynous agents.	• Androgynous agent's perceived gender	• *Androgynous agent's* *perceived gender*: - Non-androgynous agents rated first: - FP: more feminine - MP: more masculine - Androgynous agent rated first: - FP: more masculine - MP: more feminine
McDonnell et al. (2009)	• 1 MA • 1 FA • 2 neutral • 3-D • Realist • Subject	• 22 MP • 19 FP • avg age N/A	Watching a video of agents walking.	• Agents' perceived gender	• *Agents' perceived gender*: - FA (male walk): ambiguous - FA (neutral walk): female - MA (female walk): ambiguous - MA (neutral walk): male - Genderless agents: ambiguous - Genderless agents (female walk): female - Genderless agents (male walk): male - Genderless agents (neutral walk): female
McDonnell et al. (2009)	• 3 MA • 3 FA • 3-D • Realist • Subject	• 33 MP • 5 FP • avg age N/A	Watching a video of agents walking.	• Agents' perceived gender	• *Agents' perceived gender*: - FA rated 'most female': FA (bigger hips and breast size) > FA (smallest hips and breast size) - MA rated 'most male': no difference - Agents rated 'most ambiguous': FA (male walk) and MA (female walk)
Fox and Bailenson (2009)	• 4 FA • 3-D • Realist • Subject	• 43 MP • 40 FP • avg age 20.82	Participants encountered an agent (low gaze (LG) or high gaze (HG), masculine or feminine clothes) *via* virtual reality, then made judgments about them.	• Rape myth acceptance • Benevolent sexism • Hostile sexism	• *Rape myth acceptance*: masculine LG agent > feminine HG agent > masculine HG agent > feminine LG agent • *Benevolent sexism*: masculine LG agent > feminine LG agent > masculine HG agent • *Benevolent sexism*: LG agent > HG agent • *Hostile sexism*: [-1.5pt] feminine HG agent > [-1.5pt] masculine HG agent
Cloud-Buckner et al. (2009)	• 2 MA • 2 FA • 3-D • Voice • Realist • Guide	• 19 MP [-1.5pt] • 16 FP • avg age N/A	Watching an agent introducing a college campus as an online tour guide.	• Friendliness • Anger • Cooperation • Self consciousness • Adventurousness • Sympathy • Sociability • Assertiveness • Cooperation • Self consciousness • Self discipline	• *Friendliness, anger*, *cooperation, self consciousness*, *adventurousness, sympathy*: Outgoing personality: MA > FA • *Sociability, assertiveness*, *cooperation, self* *consciousness, self discipline*: Introverted personality: FA > MA
Niculescu et al. (2010)	• 1 MA • 1 FA • 1 neutral • 3-D • Text • Cartoon • Tutor	• 4 MP • 4 FP • avg age N/A	Asking an agent medical questions.	• Comfortable • Confident • Less tense • Preferred agent	• *Comfortable, confident, less* *tense*: FA > MA and androgynous agent • *Preferred agent*: FA > MA > androgynous agent
Rosenberg-Kima et al. (2010)	• 2 MA • 2 FA • 3-D • Voice • Realist • Speaker	• 119 FP • avg age 21.49	Listening to an agent describe four female engineers and the benefits of engineering.	• Interest • Self-efficacy • Utility • Agent's likeability • Fewer engineering gender stereotypes	• *Interest*: - Black FP: Black FA > others - White FP: FA > MA • *Self-efficacy, utility*, *agent's likeability*: - Black FP (Black agents): FA > MA - Black FP (White agents): MA > FA • *Fewer engineering* *gender stereotypes*: - Black FP: Black agents > White agents - White FP: FA > MA
Astrid et al. (2010)	• 1 FA • 3-D • Voice • Realist • Questioner	• 41 MP • 42 FP • avg age 37.27	Answering personal questions from an agent.	• Weak • Shy • Naive • Compassionate • Inviting	• *Weak, shy, naive, compassionate*, *inviting*: not significant
Nunamaker et al. (2011)	• 1 MA • 1 FA • 3-D • Voice • Realist • Questioner	• 53 MP • 35 FP • avg age 25.45	Answering questions from an agent simulating an airport screening.	• Power • Trustworthiness • Expertise • Likability	• *Power, trustworthiness*, *expertise*: MA > FA • *Likability*: FA > MA
Kulms et al. (2011)	• 2 MA • 2 FA • 3-D • Voice • Realist • Questioner	• 32 MP • 40 FP • avg age 35.03	Answering casual questions asked by an agent, either in a low gaze (LG) or a high gaze (HG) condition.	• Masculinity • Positive evaluation • Social presence	• *Masculinity*: HG MA > LG MA • *Positive evaluation*: FA > MA • *Social presence*: MA > FA
Brahnam and De Angeli (2012)	• 8 MA • 8 FA • 3 neutral • 2-D • Text • Cartoon • Chatbot	• 127 MP • 73 FP • avg age N/A	Chatting over text with a chatbot.	• Sexual discourse • Avg number of words about money/job, and physical appearance	• *Sexual discourse, avg number* *of words (physical appearance)*: FA > MA • *Avg number of words* *(money/jobs)*: MA > FA (among adult agents)
Ozogul et al. (2013)	• 2 MA • 2 FA • 3-D • Cartoon • Tutor	• 35 MP • 42 FP • avg age 12.83	Rating pictures of agents.	• Gender preference • Preferred agent to learn about engineering from	• *Gender preference*: - FP: FA > MA - MP: MA > FA • *Preferred agent to learn about* *engineering from*: young FA > young MA > old MA > old FA
Payne et al. (2013)	• 4 MA • 4 FA • 2-D and 3-D • Cartoon • Assistant	• 220 MP • 358 FP • avg age 35.56	Choosing an agent to assist in self-service checkouts.	• Preferred agent	• *Preferred agent*: - FP: FA > MA - MP: MA > FA
Lunardo et al. (2016)	• 2 MA • 2 FA • 2-D • Text • Realist • Assistant	• 107 MP • 147 FP • avg age N/A	Interacting with an agent over text at fnac.com.	• Attractiveness	• *Attractiveness*: - Agents (corporate clothes): FA > MA - Agents (casual clothes): FA > MA (not significant)
van der Lubbe and Bosse (2017)	• 2 MA • 2 FA • 3-D • Voice • Realist • Employee	• 55 MP • 38 FP • avg age N/A	Interacting with an agent employee to negotiate the agent's salary (assertive agent or non-assertive agent).	• Appropriate language • Sensitive • No deal reached • Persuasiveness	• *Appropriate language*: assertive FA > assertive MA • *Sensitive*: non-assertive MA > non-assertive FA • *No deal reached*: assertive MA > assertive FA > non-assertive FA > non-assertive MA (not significant) • *Persuasiveness*: assertive FA > assertive MA
Feng et al. (2017)	• 1 MA • 1 FA • 3-D • Voice • Realist • Instructor	• 31 MP • 32 FP • avg age 21.37	Acting out a scene in presence of an agent giving negative feedback.	• Inspiration • Self-blame • Helpfulness • Preferred agent	• *Inspiration, self-blame*, *helpfulness, preferred agent*: FA > MA
Mell et al. (2017)	• 1 FA • 2-D • Text • Realist • Assistant	• 241 MP • 140 FP • avg age 35.13	Answering questions from a chatbot about sensitive information, either with a picture of a real woman, a picture of a female virtual agent, or no picture.	• Reported lies • Allowing the system to do a credit check • Providing their address	• *Reported lies*: human > no presence > agent • *Allowing the system* *to do a credit check*: - FP: agent > human > no presence - MP: no presence > agent > human • *Providing their address*: - FP: equal across conditions - MP: human > agent > no presence
par Khashe et al. (2017)	• 1 MA • 1 FA • 3-D • Voice • Realist • Speaker	• 98 MP • 116 FP • avg age N/A	Requested to switch off the lights and open the window by a manager, either voice only, text only, or a virtual agent).	• Affectionate • Friendly • Likable • Persuasiveness	• *Affectionate, friendly, likable*: female (agent and voice only) > male (agent and voice only) • *Persuasiveness*: female (agent, voice only, text only) > male (agent, voice only, text only)
Kantharaju et al. (2018)	• 2 MA • 2 FA • 3-D • Voice • Realist • 2 experts • 2 motivators	• 113 MP • 92 FP • avg age N/A	Listening to a persuasive conversation about cinema between agents.	• Distant • Arrogant • Forceful • Credible • Persuasiveness	• *Distant, arrogant, forceful*, *credible, persuasiveness*: MP > FP
Akbar et al. (2018)	• 1 MA • 1 FA • 3-D • Text • Realist • Interviewer	• 158 MP • 158 FP • avg age N/A	Interviewed by an agent over text for a job in a financial firm.	• Agreeableness • Trustworthiness	• *Agreeableness*: opposite gender agent > matching-gender agent • *Trustworthiness*: matching-gender agent > opposite gender agent
Mousas et al. (2018)	• 2 MA • 3-D • Realist • Subject	• 56 MP • 16 FP • avg age 23.24	Answering questions about the agents (e.g., "Would you feel uneasy if this virtual character communicated with you?") by the experimenter while the agent walked toward the participant.	• Easiness • Comfortableness • Readiness for interaction • Likeability	• *Easiness, comfortableness*, *readiness for interaction*: MP > FP • *Likeability*: - zombie agent: MP > FP - MA: not significant
Ait Challal and Grynszpan (2018)	• 1 MA • 1 FA • 3-D • Realist • Subject	• 12 MP • 12 FP • avg age 23.6	Watched virtual agents sit in front of them (in gaze following, gaze avoidance, high direct gaze, and low direct gaze conditions). Judging their personalities.	• Neuroticism • Agreeableness	• *Neuroticism*: FA > MA • *Agreeableness (high direct* *gaze condition)*: MA > FA
ter Stal et al. (2020)	• 4 MA • 4 FA • 2-D • Cartoon • 4 experts, 4 peers	• 67 MP • 69 FP • avg age 51.36	Observing and rating 8 agents.	• Friendliness • Expertise • Authority	• *Friendliness*: FA > MA • *Expertise, authority*: MA > FA
ter Stal et al. (2020)	• 4 MA • 4 FA • 2-D • Cartoon • 4 experts, 4 peers	• 35 MP • 30 FP • avg age 67.85	Observing and rating 8 agents.	• Friendliness • Authority	• *Friendliness*: not significant • *Authority*: MA > FA
Zibrek et al. (2020)	• 2 neutral • 3-D • Realist • Subject	• 10 MP • 10 FP • avg age N/A	Pressing a button as soon as they felt uncomfortable with the distance between themselves and an agent walking toward them.	• Genderless agents' perceived gender	• *Genderless agents'* *perceived gender*: - female motions = female - male motions = male
Richards et al. (2020)	• 6 MA • 6 FA • 3-D • Voice • Realist • Assistant	• 43 MP • 146 FP • avg age 21.7	Watching 12 videos of 12 different agents introducing themselves.	• Favorite agent (before and after watching the videos)	• *Favorite agent (before)*: - FP: gender does not matter > FA > MA - MP: gender does not matter > MA > FA • *Favorite agent (after)*: Mediterranean FA > Asian FA > White FA
Nag and Yalçın (2020)	• 1 MA • 1 FA • 1 neutral • 3-D • Realist • Subject	• 41 MP • 31 FP • avg age 21.7	Looking at pictures of agents and rating them.	• Communion • Agency • Competence	• *Communion*: FA > MA (not significant) • *Agency, competence*: not significant
Esposito et al. (2021)	• 2 MA • 2 FA • 3-D • Voice • Realist • Assistant	• 22 MP • 24 FP • avg age 71.59	Watching a video of an agent talking about daycare facilities for the elderly.	• Willingness to interact with the agent • Attractiveness • Usefulness • Presentable • Professional • Of good taste • Pleasant • Original • Creative • Captivating	• *Willingness to interact with* *the agent, attractiveness, usefulness*, *presentable, professional, of good* *taste, pleasant, original*, *creative, captivating*: FA > MA
Esposito et al. (2021)	• 2 MA • 2 FA • 3-D • Voice • Realist • Assistant	• 20 MP • 25 FP • avg age 71.22	Watching a video of an agent talking about daycare facilities for the elderly. (2^nd^ experiment).	• Willingness to interact with the agent • Attractiveness • Usefulness • Presentable • Professional • Of good taste • Pleasant • Original • Creative • Captivating	• *Presentable, professional*, *of good taste, pleasant*: FA > MA • *Willingness to interact* *with the agent, attractiveness*, *usefulness, original, creative*, *captivating*: Not significant
Vilaro et al. (2021)	• 3 FA • 3-D • Voice, text • Realist • Assistant, expert	• 53 FP • avg age 60.90	Watching an agent deliver colorectal cancer screening messages.	• Trustworthiness • Expertise	• *Trustworthiness*: not significant • *Expertise*: agents (white medical coat) > agent (casual clothes)
Antonio Gómez-Jáuregui et al. (2021)	• 1 MA • 1 FA • 3-D • Voice • Realist •Interviewer	• 16 MP • 16 FP • avg age 29.95	Introducing themselves to a blurred-face virtual agent for a job interview.	• Dominance • Warmth	• *Dominance*: not significant • *Warmth*: FA (mirrored movements) > FA (random movements)
Świdrak et al. (2021)	• 1 MA • 1 FA • 3-D • Voice • Realist • Player	• 15 MP • 19 FP • avg age 25	Playing a negotiation/ decision-making game with a female and a male agent.	• Touch pleasantness • Touch awkwardness • Touch adequacy • Persuasiveness	• *Touch pleasantness*: FA > MA • *Touch awkwardness*: FP > MP • *Touch adequacy*: FA perceived as more masculine > FA perceived as less masculine • *Persuasiveness*: - MP: agents perceived as more masculine > agents perceived as less masculine - FP: depends on the offer
Świdrak et al. (2021)	• 2 MA • 2 FA • 3-D • Voice • Realist • Player	• 40 MP • avg age 23	Playing a negotiation/ decision-making game with two female and two male agents.	• Masculinity • Touch pleasantness • Touch awkwardness • Touch adequacy • Persuasiveness	• *Masculinity*: masculine FA > feminine MA • *Touch pleasantness*: FA > feminine MA • *Touch awkwardness*: feminine MA > others (not significant) • *Touch adequacy*: others > feminine MA (not significant) • *Persuasiveness*: masculine-perceived agents > feminine-perceived agents

*Articles are listed from oldest to most recent. FA, female agent; MA, male agent; FP, female participants; MP, male participants. The agents' column describes the number of agents depending on their gender, their dimension (2-D or 3-D), their appearance (realist or cartoon), and their role. The participants' column describes the number of men and women who participated in the study and the average age. In the result(s) column, “MP > FP” means it impacted more the male participants than the female participants. “FA > MA” means the female agent has more impact than the male agent. Explanations are in Section 3.4*.

## 2. Methods

### 2.1. Search Strategy

**Figure 1:**
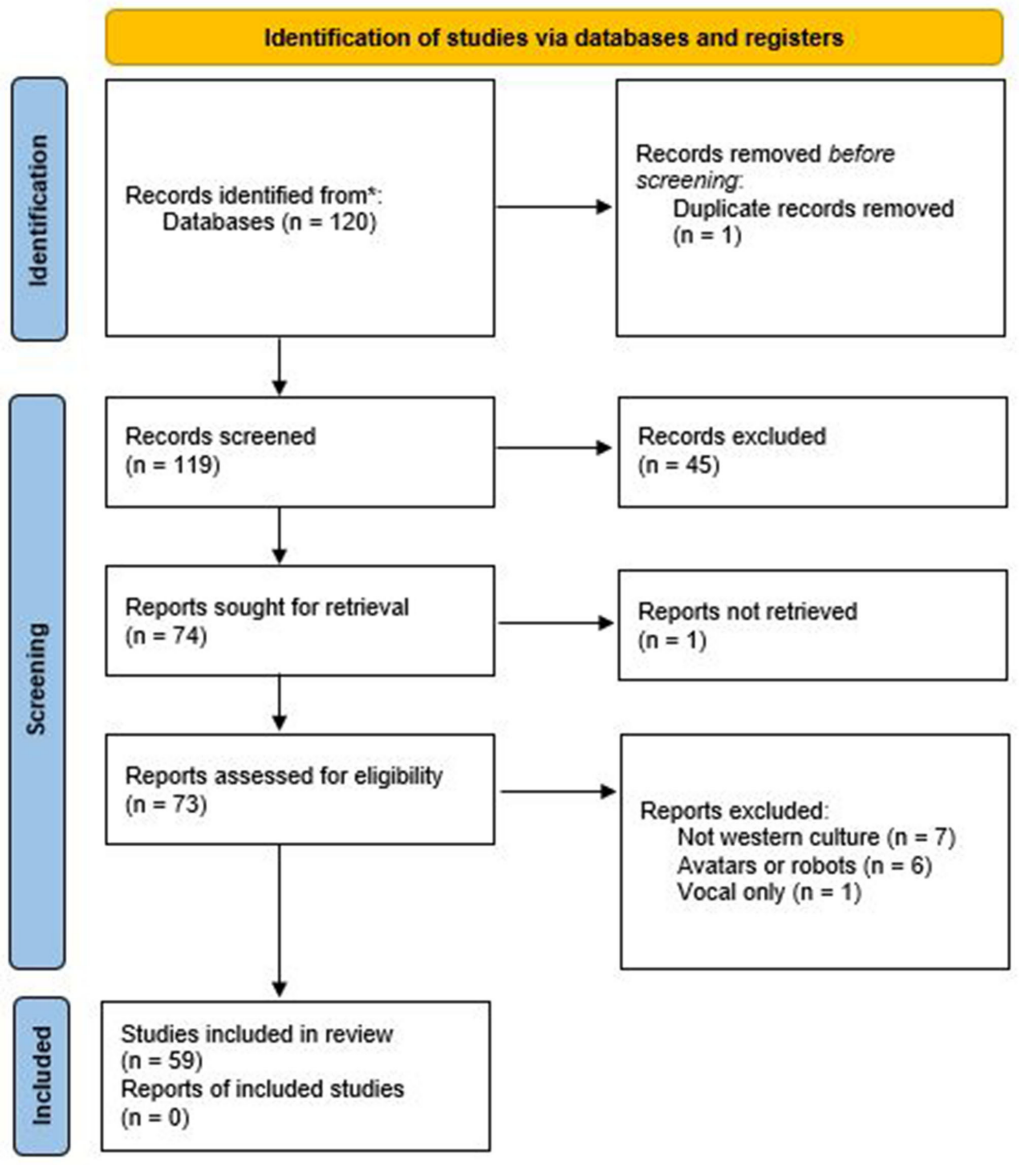
PRISMA 2020 flow diagram for new systematic reviews.

This article examines research on the impact of virtual agents' gender on learners but also more generally on users' behavior and perceptions. For this purpose, we reviewed articles from the Web of Science database over 21 years from 2000 to 2021. To collect the relevant studies, we conducted an online database search with the query gender+(“virtual agent^*^” OR “virtual character^*^”). This systematic review was conducted according to the PRISMA guidelines presented in Figure 1 (Webster and Watson, 2002) as follows: (1) scanning databases and starting with the major contributions in the leading journals, (2) reviewing the citations for the articles identified in step 1 to determine prior articles that should be considered, and (3) identifying articles citing the key articles identified in the previous steps. We used Google Scholar for the last step. A total of 120 articles were retained after following these steps.

### 2.2. Selection of Articles

From this set of articles, we selected empirical studies analyzing the effect of virtual agents' gender on users' perceptions, behaviors, and academic outcomes. We only took into account embodied virtual agents (i.e., we excluded studies on vocal assistants). We focused on Western culture and, thus, only selected papers relating to this culture. We eliminated articles only about avatars (users embodying a virtual agent) which were mainly about video games. In the end, we retained a set of 59 articles. We distinguished two types of research articles: Perceptive studies of virtual agents depending on their gender, regardless of the application domain, and research studies on the impact of gendered virtual agents in the context of a learning task.

This systematic review focuses on how virtual agents are designed, and the impact of their gender on different academic outcomes (motivation, learning, interest), but also on participants' perceptions of the agents. The research questions guiding this review are as follows:

1. Do students perceive virtual agents differently depending on their own gender and the gender of the agent?
2. Does the gender of pedagogical agents influence students' academic performance and self-evaluations?
3. Are there tasks or academic situations to which a male virtual agent is better suited than a female virtual agent, and vice versa, according to empirical evidence?
4. How do a virtual agent's pedagogical roles impact these results?
5. How do a virtual agent's appearance and interactive capacities impact these results?
6. Are androgynous virtual agents a potential solution to combatting gender stereotypes?

## 3. Systematic Review of Virtual Agents' Gender and Its Impact on Users' Perceptions and Academic Outcomes

In this section, we first review the different measures used to assess users' perceptions of agents, users' learning, and self-evaluations. We then highlight the persistence of gender stereotypes in human-machine interactions by presenting research on users' perceptions of virtual agents depending on their gender (Section 3.4). Second, we focus on pedagogical agents and discuss research that shows the effect of their gender on learners (Section 3.5).

### 3.1. Subjective Measures of Users' Perceptions of Agents'

Most of the studies used post-experience questionnaires to assess users' perceptions of virtual agents. Likert scale items were used to determine participants' stereotyped attributions of the agents, corresponding to communal traits stereotypically associated with women (e.g., affectionate, compassionate, sensitive, inviting, helpful), agency traits stereotypically associated with men (e.g., arrogant, ambitious, aggressive, courageous, and decisive), and competence traits associated more often with men (e.g., knowledgeable, intelligent, expert, credible, creative, innovative and organized) (Lee, 2003; Nunamaker et al., 2011; Feng et al., 2017; Khashe et al., 2017; van der Lubbe and Bosse, 2017; Kantharaju et al., 2018; Sczesny et al., 2018). Another questionnaire was sometimes used to determine which stereotypical gendered traits users applied to the agents (Kulms et al., 2011). This scale, the Bem Sex Role Inventory (BSRI) developed by Bem (1974), measures the construction of the gender schema of individuals, aims to highlight androgyny, and questions the usual dichotomy of female/male gendered traits stereotypically attributed to people. The BSRI consists of 20 positive items stereotypically associated with men (e.g., independent, analytical), 20 other positive items stereotypically associated with women (e.g., compassionate, loves children), and 20 other positive neutral items (e.g., tactful, reliable). The agents' gender perception was evaluated with a 5-point Likert sliding scale, e.g., with men=1, androgynous=4, and women=7 (Lee, 2003; McDonnell et al., 2009; Niculescu et al., 2009; Nag and Yalçın, 2020). Other social attitude perceptions were also assessed with Likert scale items, such as the perceived friendliness, trustworthiness, likability, and social presence of the agent (Lee, 2003; Guadagno et al., 2007; Nunamaker et al., 2011; Lunardo et al., 2016; Khashe et al., 2017; Akbar et al., 2018). Social presence is particularly important as it provides individuals with the possibility of developing a relationship or having a social interaction with one another, as they recognize each other as "social beings" (Biocca et al., 2003). Social presence is commonly defined as the sensation of being in the presence of a real person and having access to their feelings (Biocca, 1997), and can be assessed with a 5-item survey (e.g., “I feel that the person is watching me and is aware of my presence”) (Bailenson et al., 2001) and the Networked Minds Questionnaire (e.g., “The other individual didn't notice me in the room”) (Biocca et al., 2001), as used by Kulms et al. (2011).

### 3.2. Objective Measures of Learning

The impact of pedagogical agents on users' learning can be assessed by measuring users' performance in an exercise by comparing different conditions: for example, virtual agents with different behaviors (Chang et al., 2019), the presence of gendered virtual agents (Kim and Wei, 2011), or virtual agents with different genders (Kim, 2013). Performance can be measured with different problem-solving tests: using knowledge retention (using past knowledge to solve a problem, Sajjadi et al., 2020), recall (the ability to remember items, Wirzberger et al., 2019), or transfer learning (using past knowledge to solve new problems, Makransky et al., 2019). In addition to performance, researchers can also evaluate response times and effort. Effort can be measured by comparing the number of problems solved (that are not necessarily correct) in different problem-solving tests (Krämer et al., 2016). Response times correspond to the duration required to solve a problem (Hayes et al., 2010).

### 3.3. Users' Self-Evaluations

In learning situations, other more subjective measures than performance are rated using Likert-scale items. These measures include the interest in a task or a domain, (e.g., “I will take a hard sciences course as an elective,” Rosenberg-Kima et al., 2008), beliefs about the utility of a task or a domain (e.g., “I would have many good career opportunities if I was a hard science major,” Plant et al., 2009), learners' self-efficacy as in feeling capable of performing a task (e.g., “I can achieve high grades in math,” Kim and Wei, 2011), learners' self-regulation to regulate their behaviors to succeed in a task (e.g., “I kept track of my progress,” Baylor and Kim, 2004), learners' motivation assessed with the Situational Motivation Scale (SIMS) (Guay et al., 2000) which includes 16 items about the motivation to work on tasks (e.g., “Because I am doing it for my own good,” Krämer et al., 2016), learners' enjoyment (e.g., “How much did you enjoy preparing for the exam?,” Shiban et al., 2015), their perceived learning effectiveness (e.g., “I gained a good understanding of the basic concepts of the materials,” Sajjadi et al., 2020), and their mental demand to know how much mental and perceptual activity was required (thinking, deciding, calculating, etc.), e.g., “Was the task easy or demanding?” (Hart and Staveland, 1988; Pezzullo et al., 2017).

### 3.4. Evidence of the Persistence of Gender Stereotypes in Human-Machine Interactions

We have summarized the selected studies on users' perceptions of virtual agents depending on their gender in each line of the following table. We stated agents' characteristics, the number of male and female participants with their average age, tasks of the study, the observed measures, and the study's results. Some acronyms are present in this table. We used MA for Male Agent(s) and FA for Female Agent(s). In the same logic, MP is used for Male Participant(s), and FP for Female Participant(s).

The studies presented in Table 1 show that gender stereotypes persist in human-machine interactions. Users' behavior varies according to the gendered appearance of virtual agents. For example, in De Angeli and Brahnam (2006), the female virtual agent received several violent sexual propositions and even rape threats; the male virtual agent received only a few sexual propositions, none of them violent (“gently presses my lips to yours into a small kiss”), and the other sexual comments made during the interactions with the male virtual agent targeted his girlfriend. In a similar study by Brahnam and De Angeli (2012), users interacted with several pairs of female/male agents, including child agents, White agents, Black agents, and “old” agents. The female agents were the target of significantly more sexual discourse, comments on their appearance, and swear words than the male agents; this was even true for the pair of child agents. Other features of agents influenced the conversational topics, such as their age and appearance: users talked more about jobs, achievements, and money with old agents dressed in formal clothing than with any other pair of agents. However, gender stereotypes still applied to this category, since users interacted more with the older male agent about these topics than with the older female agent.

An agent's gender also has a direct influence on participants' decisions. For instance, Lee (2003) reported that users followed more advice from virtual agents when their gender stereotypically matched the topic (e.g., a female agent and cosmetics, a male agent and sports). In this study, the female virtual agent presented as particularly feminine. This result should, thus, be verified in a separate study using a female virtual agent presenting a sport-oriented appearance to determine whether these results are due solely to gender and not to the agents' presentation (clothes and make-up). In Guadagno et al. (2007), the male virtual agent was more persuasive when perceived to be computer-controlled rather than human-controlled. The opposite was true for the female virtual agent. The authors concluded that these results may have been due to gender stereotypes, specifically by the “participants” expectations for interacting with a computer being more consistent with masculine stereotypes (e.g., competent), whereas expectations for interacting with a human are more consistent with feminine stereotypes (e.g., warm)."Not only the gender of a virtual agent but even their perceived masculinity can influence participants' decisions. In a decision-making game where virtual agents made a monetary offer to male participants, the number of offers accepted was higher with the agents perceived as more masculine (Świdrak et al., 2021). The same results were obtained in a similar study for male participants; in contrast, female participants accepted more offers from the agents than male participants but were only influenced by the offer itself, not by the agents' perceived masculinity (Świdrak et al., 2021).

An agent's gender also has an impact on how users perceive the agent in terms of stereotypical traits attributed to men and women (Sczesny et al., 2018). In a study by Nunamaker et al. (2011), the male agent was perceived as more powerful, whereas the female agent was perceived as more likable. Even when male and female agents wore the same clothes, exhibited the same verbal and non-verbal behaviors, and had their faces blurred (thus lacking salient indicators of gender), the female agent was rated higher for warmth than the male agent; however, they were rated similarly for dominance, a trait typically associated with men (Antonio Gómez-Jáuregui et al., 2021). In contrast, Kulms et al. (2011), found in their main experiment that participants did not ascribe more masculine traits to the male agents nor more feminine traits to the female agents, unlike in their pretest with 14 participants using still pictures of the same virtual agents. The authors concluded that stereotyped attributions became less important when participants could interpret the behavior of the agents. However, a study by Ait Challal and Grynszpan (2018) contradicts this conclusion: the female agent was rated as less agreeable than the male agent when using high direct gaze. The authors suggested that participants were less tolerant of dominance when expressed by a female agent. Gender stereotypes associated with users' gender can also impact the ratings of virtual agents. In a study by Mousas et al. (2018), male participants reported feeling more at ease and comfortable with a zombie agent than female participants; they also liked the zombie agent more than the female participants did. The authors concluded that gender stereotypes may have influenced the results because stereotypes call for men to be calmer in the face of fear and embarrassment/disgust and to report milder emotional reactions.

Contexts stereotypically associated with one gender may also have an impact on participants' preferences as to the gender of agents: in two experiments conducted by ter Stal et al. (2020), elderly participants preferred still pictures of female agents in a healthcare context. According to the authors, this result could be due to the task—health coaching—being associated with female gender stereotypes. In addition, male agents were rated as more authoritarian and expert than female agents. In a study by Gulz et al. (2007), when virtual agents presented university programs in computer engineering, participants' interest was higher in feminine and masculine agents as compared to “neutral” agents (a less feminine female agent and a less masculine male agent). However, participants who ranked the less feminine female agent as the best presenter chose her because they believed that she could make more girls interested in computer engineering (“she seems young and nice, and I think she would make more girls interested”); and participants who ranked the feminine female agent as the worst presenter chose her because she was a woman who did not seem to belong in that context (“as I said, a woman feels more welcoming than a man, but she looked so styled, which I don't like”). These results show that gender stereotypes apply to the appearance of female agents.

In addition to context, agents' roles can also influence how users perceive them. When female agents were presented as assistants to elderly people in their daily life, participants found them to be more worth interacting with, more useful, efficient, and well designed, and more captivating, exciting, engaging, and attractive than male agents (Esposito et al., 2021). However, in a similar experiment with silent agents, the agents' gender did not affect the participants in terms of the same criteria (Esposito et al., 2021). Voices could have influenced the perceived agents' masculinity/femininity, but this was not measured in the studies. In a different study, expert agents were rated as more credible than motivational agents regardless of their gender (Kantharaju et al., 2018).

However, a recent study by Nag and Yalçın (2020) contradicts previous research on how humans perceive virtual agents depending on their gender: still pictures of male and female agents were generally rated similarly for *agency* (traits typically associated with men: *ambitious, aggressive, courageous, decisive*) and *competence* (traits typically associated with men: *creative, intelligent, innovative, organized*), but not for *communion* (traits typically associated with women: *affectionate, compassionate, sensitive, inviting, helpful*) where female agents were rated higher. A limitation of this study is that the female and male agents were quite similar in appearance. This being said, the results of the study tend to be coherent with the evolution of gender stereotypes reported by Eagly et al. (2020) for the perception of *agency* and *competence* traits perception in interpersonal interactions: the gap in *agency* and *competence* in favor of men has reduced. However, the *communion* traits are still largely attributed to women. This raises the question of whether the evolution in the perception of stereotypes in human-human interactions shown by Eagly et al. (2020) can be observed similarly in human-virtual agent interactions.

Based on the research presented above, it seems that male virtual agents are perceived as more competent, especially regarding stereotypically male-related topics. They appear as better suited to represent a pedagogical virtual tutor in STEM fields (Science, Technology, Engineering, and Mathematics) since these fields are perceived as masculine (Makarova et al., 2019). In the next section, we focus more specifically on research on pedagogical agents and the impact of their gender on users' academic outcomes.

### 3.5. The Effect of Virtual Agents' Gender on Academic Outcomes

We have summarized the selected studies on the impact of gendered virtual agents in the context of a learning task in each line of the following table. We stated agents' characteristics, the number of male and female participants with their average age, tasks of the study, the observed measures, and the study's results. Some acronyms are present in this table. We used MA for Male Agent(s) and FA for Female Agent(s). In the same logic, MP is used for Male Participant(s), and FP for Female Participant(s).

Various studies on virtual learning environments (Table 2) have reported that the gender of a pedagogical agent may have an impact on the learning performance of users. In a recent article, Makransky et al. (2019) showed that young girls performed better on scientific tasks (in terms of learning and transfer learning) when taught by a virtual female scientist than by a virtual drone. The opposite was true for boys. The researchers argued that boys identified with the drone, while girls identified with the female agent. However, research opposing human-like vs. robot-like agents does not take into account other factors that may influence how girls learn. In a study by Shiban et al. (2015), female learners were more motivated and interested in math when trained by a female agent as compared to a male agent. However, they obtained better results with the male agent, which may be explained by their perception of the agents' appearances: the male agent was older and wore a tie, while the female agent was young and pretty. According to the authors, the participants' performance improved because the male agent was perceived as an expert, and virtual agents perceived as experts have been shown to improve learners' performance (Baylor and Kim, 2004). The researchers also concluded that the female participants' motivation and interest improved with the female agent because there were more female participants in the study and because of the agent's similarity (in age and gender) to them, in line with the “similarity hypothesis” (also found in Rosenberg-Kima et al. 2008). This argument is supported by Bandura's social cognitive learning theory: people often learn by imitating people whom they perceive as similar (or superior: higher in rank or status) to them and who are, therefore, accepted as social role models (Bandura and National Inst of Mental Health, 1986). This theory also bears out in a study by Plant et al. (2009), where a female agent raised participants' self-efficacy by delivering a message on the benefits of engineering, resulting in better performance and more interest in math.

**Table 2:** Summary of research studies on the impact of gendered virtual agents in the context of a learning task.

**Reference(s)**	**Agent(s)**	**Participant(s)**	**Task(s)**	**Measure(s)**	**Result(s)**
Moreno et al. (2002)	• 2 MA • 2 FA • 3-D • Voice • Realist • Tutor	• 12 MP • 27 FP • avg age 20	Watching a video of a virtual agent giving a course, taking a multiple-choice test.	• Performance • Perceived masculinity, femininity	• *Performance*: MA > FA • *Perceived masculinity, femininity*: - FA: very feminine - MA: masculine
Baylor and Kim (2004)	• 4 MA • 4 FA • 2-D, 3-D • Voice • Realist, cartoon • Tutor	• 94 MP • 218 FP • avg age 20.54	Creating an instructional schedule with a virtual agent's help.	• Self-efficacy • Self-regulation • Knowledgeability • Intelligence • Learning	• *Self-efficacy, self-regulation*, *knowledgeability, intelligence*: MA > FA • *Learning*: not significant
Baylor and Kim (2004)	• 6 MA • 6 FA • 2-D, 3-D • Voice • Realist, cartoon • Expert, motivator, mentor	• 89 MP • 140 FP • avg age 19.39	Creating an instructional planning with a virtual agent's help.	• Knowledgeability • Intelligence • Learning • Self-regulation • Self-efficacy	• *Knowledgeability, intelligence*: MA > FA • *Learning, self-regulation:* not significant • *Self-efficacy*: FA > MA
Moreno and Flowerday (2006)	• 5 MA • 5 FA • 2-D • Voice • Realist • Tutor	• 21 MP • 59 FP • avg age 26.88	Watching a video of a course taught by a virtual agent, taking a test.	• Helpfulness • Motivation • Selected agent • Learning	• *Helpfulness, motivation, learning*: not significant • *Selected agent*: matching-gender agent = opposite gender agent
Kim et al. (2007)	• 1 MA • 1 FA • 3-D • Voice • Realist • Companion	• 11 MP • 45 FP • avg age 20.71	Creating a course on economic concepts with a virtual agent's help.	• Facilitating learning • Engaging • Human-like • Learning (recall)	• *Facilitating learning, engaging*, *human-like*: MA > FA • *Learning (recall)*: not significant
Plant et al. (2009)	• 1 MA • 1 FA • 3-D • Voice • Realist • Speaker	• 45 MP • 61 FP • avg age 13.63	Listening to a story about four female engineers and the benefits of engineering, either delivered by an agent or voice-only. Taking a math test.	• Interest • Utility • Self-efficacy • Performance • Fewer engineering gender stereotypes	• *Interest, utility*: FA > MA and no agent • *Self-efficacy*: MA and FA > no agent • *Performance*: FA > MA • *Fewer engineering* *gender stereotypes*: - MP: agents > no agent - FP: FA and no agent > MA
Hayes et al. (2010)	• 1 MA • 1 FA • 3-D • Voice • Realist • Observer	• 35 MP • avg age 19.77	Controlling an avatar (1^st^ or 3^rd^ person view) while taking a math test, in the presence of a male or female agent, or without an agent.	• Social presence • Performance • Response times	• *Social presence*: MA > FA and no agent • *Performance, response times*: - 1^st^ person: no agent and MA > FA - 3^rd^ person: FA > no agent and MA
Kim and Wei (2011)	• 2 MA • 2 FA • 3-D • Voice • Realist • Tutor	• 110 MP • 100 FP • avg age 15.93	Taking a math test without an agent, watching an agent explaining the lessons, resolving math problems with the agent (training), taking a 2^nd^ math test without an agent.	• Selected agent • Performance	• *Selected agent*: matching gender and matching ethnicity agents > others • *Performance*: everyone improved
Silvervarg et al. (2013)	• 1 MA • 1 FA • 1 neutral • 2-D • Text • Cartoon • Tutee	• 46 MP • 37 FP • 12–14 years old	Interacting with an androgynous virtual tutee on a math lesson, then with either a female or a male virtual tutee.	• Perceived androgyny • Preferred agent as tutee • Preferred agent as chat partner	• *Perceived androgyny*: androgynous agent = androgynous • *Preferred agent as tutee*: - FP: androgynous agent > MA and FA - MP: androgynous agent > MA and FA (not significant) • *Preferred agent as chat partner*: - FP: androgynous agent > MA - MP: androgynous agent > FA
Kim and Lim (2013)	• 2 FA • 3-D • Voice • Realist • Tutor	• 64 MP • 56 FP • avg age 15.93	Taking a math test without an agent, learning lessons with or without an agent, resolving math problems with or without an agent (training), taking a 2^nd^ math test without an agent.	• Performance • Self-efficacy	• *Performance*: everyone improved • *Self-efficacy*: - FP: agent present > no agent - MP: no increase
Kim (2013)	• 1 MA • 1 FA • 1 neutral • 2-D, 3-D • Voice, text • Cartoon • Tutor	• 68 MP • 73 FP • avg age N/A	Answering questions about a text asked by a virtual agent.	• Text comprehension	• *Text comprehension*: - FP = MP - FP: MA and FA > robot agent - MP: MA > FA and robot agent - Ethnic-minority participants: - MA: peer agent > teacher agent - FA: teacher agent > peer agent - Caucasians participants: - MA and FA: not significant • *Positive attitudes to learn math*: - Ethnic-minority participants: - MA: peer agent > teacher agent - FA: teacher agent > peer agent - Caucasians participants: - MA and FA: not significant
Krämer et al. (2016)	• 2 MA • 2 FA • 3-D • Voice • Realist • Motivating interviewer	• 60 MP • 68 FP • avg age 23.85	Taking a math test without an agent, then taking a math test with an agent present explaining the procedure.	• Motivation • Sense of rapport • Performance	• *Motivation, sense of rapport*: not significant • *Performance*: - FP and rapport agent: MA > FA - MP and rapport agent: FA > MA
Li et al. (2016)	• 1 MA • 1 neutral • 3-D • Voice • Realist • Tutor	• 20 MP • 20 FP • avg age 20.48	Watching an agent present slides on courses about Human-Computer Interaction.	• Learning	• *Learning*: - MP: agent robot > real human (male) > MA > still image of a robot - FP: no differences
Jeong et al. (2017)	• 1 MA • 1 FA • 3-D • Voice • Realist • Instructor	• 54 MP • 63 FP • avg age 20.94	Listening to negative feedback from an instructor agent while acting out a scene. Reproducing the scene with the instructor agent and a student agent (no feedback).	• Moving forward • Moving backward	• *Moving forward*: - FP: FA > MA - MP: MA > FA • *Moving backward*: - FP: MA > FA - MP: FA > MA
Pezzullo et al. (2017)	• 1 FA • 3-D • Voice • Realist • Companion	• 54 MP • 63 FP • avg age 13.30	Playing a game about biology courses with a virtual agent's help.	• Mental demand • Engagement with the agent • Performance	• *Mental demand, engagement* *with the agent*: FP > MP • *Performance*: FP = MP
Wirzberger et al. (2019)	• 1 MA • 3-D • Voice • Realist • Instructor	• 27 MP • 35 FP • avg age 69.03	Memorizing a word list after taking a memory training course led by an agent.	• Learning (recall)	• *Learning (recall)*: FP > MP
Makransky et al. (2019)	• 1 FA • 1 neutral • 3-D • Voice • Realist • Tutor	• 33 MP • 33 FP • avg age N/A	Watching a virtual agent teaching laboratory safety, taking tests.	• Social presence • Learning (recall and transfer-learning)	• *Social presence*: - FP: FA = drone agent - MP: FA > drone agent • Learning (recall and *transfer-learning)*: - FP = MP - FP: FA > drone agent - MP: drone agent > FA
Chang et al. (2019)	• 1 MA • 3-D • Voice • Realist • Instructor	• 76 FP • avg age N/A	Controlling either a male or a female avatar, learning how to solve arithmetic problems from a male agent (either a dominant or a non-dominant agent, based on his body posture), solving problems without the agent present.	• Learning (recall and performance)	• *Learning (recall and performance)*: - non-dominant agent > dominant agent - No significant effect of avatar's gender
Sajjadi et al. (2020)	• 1 MA • 1 FA • 3-D • Voice • Realist • Instructor	• 8 MP • 4 FP • avg age 19.6	Observing geologic formations in a virtual environment, answering questions asked by an agent.	• Perceived learning effectiveness • Learning • Leadership • Friendliness • Social and spacial presence	• *Perceived learning* *effectiveness*: FA > MA • *Learning*: not significant • *Leadership, friendliness, social* *and spacial presence*: FA > MA (not significant)
Spilioto-poulos et al. (2020)	• 1 FA • 3-D • Voice • Realist • Tutor	• 24 MP • 16 FP • avg age 20	Learning how to use argumentation, how to be empathetic to the needs of others, how to reach agreements through negotiation with a virtual agent.	• Self-efficacy • System easiness • Helpfulness • Learning	• *Self-efficacy*: not significant • *System easiness*: FP > MP • *Helpfullness*: MP > FP • *Learning*: not significant (increase overall)

*Articles are listed from oldest to most recent. FA, female agent; MA, male agent; FP, female participants; MP, male participants. The agents' column describes the number of agents depending on their gender, their dimension (2-D or 3-D), their appearance (realist or cartoon), and their role. The participants' column describes the number of men and women who participated in the study and the average age. In the result(s) column, “MP > FP” means it impacted more the male participants than the female participants. “FA > MA” means the female agent has more impact than the male agent. Explanations are in Section 3.5*.

However, other research has demonstrated a positive effect of male agents as compared to female agents in pedagogical tasks. For instance, in two experiments conducted by Baylor and Kim (2004), a virtual agent helped participants create a schedule. The agent's gender did not impact learning but did affect self-efficacy, which increased more in the first experiment with the male agent than the female agent; the contrary occurred in the second experiment for both male and female participants. The researchers suggested that there was a bias in the first experiment, as participants rated the male agent as more interesting and useful than the female agent. In the second experiment, participants viewed the female agent as less expert and knowledgeable than the male agent, despite receiving the same instructions from both agents; some research has indicated that agents perceived as less intelligent could lead to greater self-efficacy (Baylor and Kim, 2005). In a similar experiment by Kim et al. (2007), the researchers introduced a female and a male pedagogical agent to help students design an e-learning course, which included creating a schedule. Students working with the male agent rated him higher on facilitating learning, being engaging, and being human-like than students working with the female agent. Notably, the male agent had a more positive impact than the female agent on the participants' interest and learning in terms of recall (the ability to remember what the agent said during the task).

Other factors may also come into play in studies on the impact of virtual agents on learning. In Moreno et al. (2002), participants watched a video of a virtual agent presenting a course on blood pressure, followed by a multiple-choice test. The results of this study suggest that the participants learned more from the male agent than from the female one. The researchers suggested that this might be because the female tutor did not conform to the stereotype of men as teachers. The first study showed that participants in this experiment perceived the female agent as very feminine, while the male agent was found to be very masculine. This may be due to a difference in the participants' interpretation of the female agent as being “too feminine” to be suitable for the role of tutor. The study did not address how participants perceived the agents' expertise or seek to determine any possible interactions between perceived expertise, the perceived agent's femininity, and performance on the test. Gender, while important, must be taken into account in combination with other features. For instance, Krämer et al. (2016) analyzed the impact of pedagogical agents' gender and their behavior on adults' motivation, effort, and performance in math. They found that the simple presence of a female virtual agent in a learning situation did not increase women's motivation and learning. However, when the agent displayed human-like non-verbal behavior by aligning with the participants' non-verbal behavior (Gratch et al., 2007), the participants' performance and effort improved. This kind of behavior, called rapport, is defined in social psychology as the establishment of a positive relationship between interactants by way of a positive attitude (e.g., acquiescence, smiles), mutual attention (e.g., mutual gaze), and coordination of behaviors (e.g., synchrony, mimicry) (Tickle-Degnen and Rosenthal, 1990). This research shows the importance of the pedagogical agents' behavior combined with their gender as providing a positive impact on academic outcomes. Agents' behavior is especially important as it could negatively impact learners' academic outcomes, as shown in an experiment by Chang et al. (2019) where a male “dominant” pedagogical agent impaired female participants' performance and recall in arithmetic problems, compared to a male “non-dominant” agent.

The research presented above highlights the importance of pedagogical agents' gender on learning. Different studies appear to yield contradictory results, on one hand, that learning improves when virtual agents' gender matches the learner's, but on the other hand that male virtual agents could be better suited to improving learning. Interestingly, Section 3.4 shows that male agents are perceived as more competent than female agents, and users follow more advice from a male agent than a female one on topics stereotypically perceived as masculine. However, the studies featuring a female agent in STEM fields (Science, Technology, Engineering, and Mathematics) presented in this section show that female agents have a positive influence on academic outcomes: they improve learning, self-efficacy, interest, and motivation, despite the fact that STEMs are perceived as masculine (Makarova et al., 2019).

## 4. Discussion

### 4.1. The Question of Pedagogical Agents' Gender

Based on the research presented above, one could surmise that, in general, male pedagogical agents are better suited to improving academic outcomes than female agents. However, systematically relying on male pedagogical agents could have an adverse impact: for instance, designing only male agents for learning purposes in STEM fields could strengthen gender stereotypes. As highlighted by West et al. (2019), the gender bias of interactive systems not only perpetuates stereotypes but also reinforces and extends them. The stereotypes modeled through interactive systems generate behaviors that go beyond the sphere of the virtual environment by conveying a harmful image of women. For instance, in a study by Dill et al. (2008), still pictures of men and women in suits or male and female characters acting in highly stereotypical ways were shown to participants. Male participants exposed to negative female stereotypes were significantly more tolerant of a real-life instance of sexual harassment and exhibited greater rape myth acceptance. As for representation in STEM fields, as noted by Sansone (2019), the lack of female role models can lead female students to believe that men are better than women in STEM fields. The lack of *virtual* female role models in virtual learning environments may have the same impact. Accordingly, more STEM experts represented with virtual female characters could help decrease gender stereotypes in STEM fields.

Some research has explored the use of androgynous virtual agents to counter gender stereotypes. In earlier studies, participants tended to apply the labels of “man” or “woman” to androgynous agents. For instance, in Niculescu et al. (2009), participants classified androgynous virtual characters as male or female, depending on the participants' gender and other parameters such as which virtual characters they had seen before. Even for genderless agents such as a wooden mannequin, the participants perceived their gender depending on how they perceived their walking motions (McDonnell et al., 2009). Recent research has shown more promising results in terms of gender stereotypes. In Nag and Yalçın (2020), results for androgynous agents show a linear trend that positions their scores for the perceived agency, communion, and competence in between those for female and male agents. The authors, thus, believe that androgynous agents could help mitigate male and female stereotypes. Although participants in their first experiment tended to believe that the androgynous virtual agents were men, when the authors modified the agents in question for their main experiment, participants correctly perceived them as androgynous after reading a definition of an androgynous agent.

What about androgynous pedagogical agents in an educational context? Silvervarg et al. (2013) supposed, but with caution, that students could identify with an androgynous agent by ascribing their own gender to them, thus making them a suitable role model. Indeed, in their experiment with children aged 12-14, participants perceived an androgynous pedagogical agent as not clearly a boy nor clearly a girl, but they generally assigned themselves a gender to their androgynous virtual tutee, boy or girl. The authors supposed students could, therefore, have more freedom to construct and ascribe gender, as their pedagogical agent's gender choice is personal rather than imposed. They also supposed androgynous agents could diminish gender stereotypes, as their appearances are genderless. Applying our own gender to an androgynous agent to make them a suitable role model is an interesting hypothesis. However, we do not know what the gender participants applied to the androgynous agent or why. More research on androgynous agents has to be done in an educational context to help determine, e.g., whether androgynous agents are perceived as masculine, feminine, neutral, man, woman, or genderless depending on the context and the role of the agent. Since STEM fields are considered masculine fields (Makarova et al., 2019), participants could perceive an androgynous agent as a man, even though they could perceive them as not clearly a boy nor a girl in terms of appearance. This could reinforce the stereotype of STEM fields as more suitable for men than women. Agents' role is also particularly important, as Brahnam and Weaver (2015) stated there are more female assistant agents than male ones. They showed the example of a webpage that provides virtual agents, four of the five virtual agents are female and they assist people at airports or serve as talking mannequins for fashion and museum exhibits. The male agent was called a “virtual doctor” and provided health tips and hospital information. We can emit the hypothesis that one could perceive androgynous virtual assistants as women, hence reinforcing gender stereotypes. For this research on androgynous virtual agents, we recommend measuring how participants feel toward the androgynous agents, as not being able to perceive someone as a man or a woman may induce insecurity and unease in some people (Nass and Brave, 2005).

### 4.2. Virtual Agents' as Social Role Models in Learning Environments

Some research, though still very limited, has explored the use of virtual agents to increase learners' performance and interest in mathematics. For example, Rosenberg-Kima et al. (2008) showed the effectiveness of a female virtual agent engineer in interesting women in STEM fields. In a video, the agent, who was similar in gender to the participants (who were all women), presented a story about successful female role models in STEM fields. This led to a change in participants' attitudes toward science, as shown with a 7-point scale questionnaire. Women in the female virtual agent condition were less likely to endorse traditional STEM stereotypes than those in the male virtual agent condition and were more likely to believe that women could succeed in STEM fields. Gender stereotypes still persisted: the participants were slightly more likely to believe in STEM usefulness with a male virtual agent engineer. In a similar study by Plant et al. (2009), male and female participants performed better and were more interested in engineering after interacting with a female agent, as their self-efficacy and their ratings about STEM usefulness improved. Interestingly, male participants were less likely to endorse traditional STEM stereotypes in the presence of an agent, male or female; but female participants were less likely to endorse traditional STEM stereotypes with a female agent or without any agent, than with a male one. Another similar study by Rosenberg-Kima et al. (2010) showed that Black virtual agents had a more positive impact on STEM interest and STEM gender stereotypes for Black women, whereas female virtual agents (Black or White) had a more positive impact on White women on the same criteria. This research shows the importance of other factors, such as virtual agents' ethnic background, performance, and interest in math.

Finally, several studies have shown that pedagogical agents used as learning companions can simulate social interactions (Kim and Baylor, 2006) and the potential impact of a virtual agent's gender on education. However, only few studies have explored the use of a virtual pedagogical companion to counteract the effects of Stereotype Threat (refer to Introduction). Research on Social Cognition has shown the positive impact of social role models to counteract ST effects (Bagès et al., 2016). Studies have shown that female participants do not immediately see female scientists as potential role models simply by interacting with them; they begin to perceive female scientists as role models when they establish personal connections with them (Buck et al., 2008). In the field of virtual agents, virtual rapport has been studied as a means to create this type of relationship between virtual agents and users (Gratch et al., 2007). As reported by Krämer et al. (2016), the mere presence of a female agent did not improve participants' performance and effort. However, when agents were able to create a virtual rapport, participants' performance and effort were shown to improve.

Based on the research presented above, not only is the gender of pedagogical agents important, but so is their behavior (Krämer et al., 2016; Chang et al., 2019), their role (Baylor and Kim, 2004; Kim, 2016), and their ethnicity (Rosenberg-Kima et al., 2010; Kim, 2016). Girls may see a female pedagogical agent as a role model who influences their motivation to exert effort to learn (Shiban et al., 2015). A study by Pfeifer and Lugrin (2018) shows that female social robots can be role models to female students: female students learned better with a female robot in a stereotypically masculine domain. Virtual characters can be used to embody social models and, thus, change the learner's attitudes and motivation; as described earlier, a female role model who succeeds in math can reduce Stereotype Threat effects. Combining research on social cognition and virtual agents, we recommend counteracting Stereotype Threat effects for girls and women in math by using a virtual agent representing a hardworking female social role model (Bagès et al., 2016) able to establish rapport with the learners (Gratch et al., 2007), of similar ethnicity to the learners (Kim, 2016) and slightly older than them (Bagès and Martinot, 2011). When the role model is younger or the same age as the learners, they can lose motivation by feeling unable to match their role model's achievements; if the model is too old, they will not identify with them. A pedagogical agent should, thus, embody the role of a knowledgeable and motivational person; this has been demonstrated by student preferences and by the proven positive impact these types of agents have on education (Kim and Baylor, 2016).

### 4.3. Improved Learning or Better Inclusion?

An ethical tension between two competing goals arises in all domains: skill learning (where the research presented above favors the use of a male virtual agent), vs. better inclusion of girls and women (*via* the use of a female virtual character embodying a successful role model in the domain). Prior research is not robust enough to prove the superiority of a male agent in all fields and for all audiences. Some questions remain unanswered in the literature, to our knowledge: Would using the same androgynous character but presented as male, female, or neutral by the experimenter have an impact on academic outcomes in scientific or other domains? What would be the impact of systematically using a virtual agent of the same gender as the learners?

Regarding the second question, using only successful male models in mathematics with boys could reinforce gender stereotypes. Women are aware of the negative stereotype about their mathematical skills that create a hostile environment for them. Research by Stokes et al. (1995) reported that when women find a friendly environment, they are more likely to stay employed. One solution to reconciling the two goals, at least in STEM fields, would be to use successful female role models to explain how they managed to perform well: in Bagès et al. (2016), students took a math test after reading a story about a social model, female or male depending on the condition. The stories differentiated between models: the hardworking model put in the effort and spent time learning his or her lessons to perform well, the gifted model was naturally good at math, and the neutral model gave no explanation for his or her success. Girls' performance increased with the hardworking model: they performed at the same level as boys, whether the model was a boy or a girl. There was no impact on boys' performance. In contrast, in a similar study, boys' performance also increased with a hardworking model, regardless of gender (Bagès and Martinot, 2011). Furthermore, when the role model did not explain his or her success in math, both girls' and boys' scores improved with a female role model. As the lack of female role models may lead female students to believe that men are better than women in STEM fields Sansone (2019), it could be interesting to combine the results of (Bagès et al., 2016) and our hypothesis that a successful *virtual* female role model in STEM fields could help mitigate gender stereotypes. Female virtual agents who act as successful social models in mathematics and explain how they succeeded through their effort and hard work may be a potential solution to counteracting Stereotype Threat effects. Another question then arises: in the long term, what would be the impact of presenting only these kinds of female virtual agents to boys?

## 5. Conclusion

In this article we have presented a systematic review of research on perceptive studies of virtual agents depending on their gender, regardless of the application domain; and on the impact of gendered virtual agents in the context of a learning task. Each study has been performed in a specific learning context with specific pedagogy, design of the virtual environment, duration of the interaction, modality of interaction, physical environment, etc. These elements of context may have an impact on the users' perceptions and learning outcomes. The limitation of this article is that we have not considered all these contextual specificities. Further analysis could take into account these contextual elements to provide a more global view of the impact of virtual agents' gender in academic learning. Nevertheless, the present systematic review enables us to draw some conclusions by answering each question stated in Section 2.2.

###  Do Students Perceive Virtual Agents' Differently Depending on Their Own Gender and the Gender of the Agent?

As individuals communicate with virtual agents by applying social rules and expectations as social beings (Nass and Moon, 2000), it is not surprising that they also apply gender stereotypes to virtual agents and their interactions with them (refer to Section 3.4). Female virtual agents are usually seen as less expert, less knowledgeable, and less powerful than male virtual agents (Baylor and Kim, 2004; Nunamaker et al., 2011), and they are also usually perceived as more likable and attractive than male virtual agents (Nunamaker et al., 2011; Lunardo et al., 2016). Those perception differences can even affect people's decisions (Lee, 2003; Świdrak et al., 2021).

Given the empirical results, we propose to respond simultaneously to question 2 (*Does the gender of pedagogical agents influence students' academic performance and self-evaluations?*) and question 3 (*Are there tasks or academic situations to which a male virtual agent is better suited to than a female virtual agent, and vice versa, according to empirical evidence?*). The review conducted in this article could lead to the belief that male pedagogical agents are better suited than female agents to improve academic outcomes, especially in male-dominated scientific fields like STEM fields (Makarova et al., 2019). However, research also shows that female pedagogical agents can improve learners' performances in these fields (Rosenberg-Kima et al., 2008; Plant et al., 2009). Some studies have shown that using female virtual agents as social role models can increase female participants' self-efficacy (Rosenberg-Kima et al., 2008), and both male and female participants' interest (Plant et al., 2009; Rosenberg-Kima et al., 2010), and performances (Plant et al., 2009). These results are especially relevant to addressing Stereotype Threat effects, a phenomenon that illustrates how and why female students' performance in math can be impaired by gender stereotypes (Spencer et al., 1999). Previous research showed that stereotype threat effects can be counteracted by introducing a female positive role model who looks like a learner so that they can identify with her (Bagès et al., 2016). Such a positive role model could be embodied by a virtual pedagogical agent (Rosenberg-Kima et al., 2008), and used to reduce stereotype threats in STEM fields.

###  How Do a Virtual Agent's Pedagogical Roles Impact These Results?

Individuals tend to listen more to agents whose gender stereotypically matches the context or gender roles such as female virtual agents with cosmetics and male virtual agents with sports (Lee, 2003), female virtual agents in contexts involving social influence (Khashe et al., 2017), and female virtual agents in an assistant role (Esposito et al., 2021). The role of a virtual pedagogical agent has been studied in the academic context, using *expert, motivator*, and *mentor* agents (Baylor and Kim, 2004). Expert agents are older than students, authoritative, strictly informative, and knowledgeable. Motivator agents are enthusiastic and not seen as particularly knowledgeable, they are mostly used to elicit motivation. As for mentor agents, they are slightly older than students, are knowledgeable, and are also used to elicit motivation. They are a mix of expert agents and motivator agents (Baylor and Kim, 2005). Researchers should take agents' roles into account when designing a pedagogical agent, as a female pedagogical agent designing as a mentor agent can improve learners' performance (Plant et al., 2009), but a female pedagogical agent designing as a motivator agent may not be effective on learners' performance (Shiban et al., 2015). In conclusion, the impact of the virtual agent's gender depends on several aspects related to the role of the agent. This role is indeed reflected through for instance the appearance but also the discourse of the agent.

###  How Do a Virtual Agent'S Appearance and Interactive Capacities Impact These Results?

The topics of individuals' interactions with virtual agents differ depending on their gendered appearance: in studies by De Angeli and Brahnam (2006) and Brahnam and De Angeli (2012), female virtual agents received significantly more violent sexual propositions, more rape threats, more comments on their appearance, and more swear words compared to male virtual agents who received few sexual propositions, most of them targeting their girlfriends. Moreover, the degree of perceived masculinity and femininity can influence men's decisions, as shown in Świdrak et al. (2021) where male participants were persuaded more by masculine agents than feminine agents, regardless of the agents' gender. Agents' appearance can influence their perceived role, as an old agent wearing a tie could be perceived more as an expert than a young agent (Shiban et al., 2015). As seen in the question above, the agents' role is important in academic situations. In addition to agents' roles and appearance, research has shown the importance of a positive relationship between learners and pedagogical agents (Krämer et al., 2016). This research tends to show that a female social model embodied by a pedagogical agent able to establish a positive relationship with learners may counteract Stereotype Threat effects and, thus, improve women's performance, interest, and self-efficacy in mathematics.

###  Are Androgynous Virtual Agents' a Potential Solution to Combatting Gender Stereotypes?

This question is quite difficult to answer as to our knowledge, few studies have explored the use of androgynous virtual agents to counter gender stereotypes in the academic context. Silvervarg et al. (2013) used an androgynous pedagogical agent in an educational context and showed participants, although evaluating the agent as “not clearly a boy nor a girl,” tend to ascribe a binary gender (boy or girl) to the agent. The authors, thus, cautiously supposed that students could ascribe their own gender to an androgynous agent, thus giving them more freedom and making the agent a suitable role model, known to be beneficial for academic outcomes. However, the results did not show what gender participants ascribe to the agent, nor why. The way individuals ascribe gender to an androgynous or a genderless agent should be studied more. In particular, does this gender attribution depend on the context or agents' role? Since STEM fields are considered masculine fields (Makarova et al., 2019), participants could think androgynous agents are men. This could reinforce the stereotype of STEM fields as more suitable for men than women. As for agents' roles, there are more female virtual assistants than male ones (Brahnam and Weaver, 2015). Some developers admitted female virtual assistants are usually used because they evoke gender stereotypes: women are expected to serve, help, and nurture others. Androgynous virtual assistants could then be considered women, and reinforce harmful stereotypes about women. Researchers and developers who want to use androgynous agents to combat gender stereotypes should be very careful, as the opposite effect can occur.

## Data Availability Statement

The original contributions presented in the study are included in the article/supplementary material, further inquiries can be directed to the corresponding author/s.

## Author Contributions

MA wrote the article. MO and IR supervised the article and corrected it. All authors contributed to the article and approved the submitted version.

## Funding

This work was carried out within the pilot center Ampiric, funded by the French State's Future Investment Program (PIA3/France 2030) as part of the “Territories of Educational Innovation” action.

## Conflict of Interest

The authors declare that the research was conducted in the absence of any commercial or financial relationships that could be construed as a potential conflict of interest.

## Publisher's Note

All claims expressed in this article are solely those of the authors and do not necessarily represent those of their affiliated organizations, or those of the publisher, the editors and the reviewers. Any product that may be evaluated in this article, or claim that may be made by its manufacturer, is not guaranteed or endorsed by the publisher.
