# Corrigendum: The impact of pedagogical agents' gender on academic learning: a systematic review

**DOI:** 10.3389/frai.2023.1302277

**Published:** 2023-10-11

**Authors:** Marjorie Armando, Magalie Ochs, Isabelle Régner

**Affiliations:** ^1^Aix Marseille Univ, CNRS, LIS UMR 7020, Marseille, France; ^2^Aix Marseille Univ, CNRS, LPC, Marseille, France; ^3^Pôle pilote Ampiric, Institut National Supérieur du Professorat et de l'Éducation, Aix-Marseille Université, Marseille, France

**Keywords:** virtual agent, gender, pedagogical agent, learning environment, gender stereotypes, systematic review

In the published article, there was an error in affiliations 1 and 3. Instead of “Aix Marseille Univ, Université de Toulon, CNRS, LIS, Marseille, France,” it should be “Aix Marseille Univ, CNRS, LIS UMR 7020, Marseille, France.” Instead of “Pôle pilôte Ampiric, Institut National Supérieur du Proffessorat et de l'Éducation, Aix-Marseille Université, Marseille, France,” it should be “Pôle pilote Ampiric, Institut National Supérieur du Professorat et de l'Éducation, Aix-Marseille Université, Marseille, France.”

In the published article, there was also an error in the Funding section, the Funding statement was not included. The correct Funding statement appears below.

